# Portuguese Version of the Ageing Attitudes Questionnaire (AAQ): Validation of the Psychometric Properties

**DOI:** 10.3390/ijerph192416778

**Published:** 2022-12-14

**Authors:** Margarida Pedroso de Lima, Paulo Martins, António-José Gonzalez

**Affiliations:** 1 Center for Research in Neuropsychology and Cognitive and Behavioral Intervention (CINEICC), Faculty of Psychology and Educational Sciences, University of Coimbra, 3004-531 Coimbra, Portugal; 2 Laboratory of Sport Psychology, Faculdade de Motricidade Humana, Universidade de Lisboa, 1499-688 Cruz-Quebrada, Portugal; 3 APPsy-CI, Applied Psychology Research Center, Capabilities & Inclusion ISPA—University Institute of Psychological, Social and Life Sciences, 1149-041 Lisbon, Portugal

**Keywords:** ageing, attitudes, subjective well-being, positive affect, psychometric properties

## Abstract

The Ageing Attitudes Questionnaire—AAQ was validated for the Portuguese population to understand the importance of attitudes towards old age and their impact on the subjective well-being of older adults. A sample of 400 subjects (from 18 to 93 years) answered a socio-demographic questionnaire, and the AAQ, composed of three subscales (psychosocial losses, physical change, and psychological growth). The CFA confirmed the tri-factorial structure with very good adjustment of the model to the data, with the Cronbach alpha of the total scale scoring 0.84 and ranging from 0.65 to 0.77 for each factor. A total of nine items were omitted for poor factor loadings (<0.50), namely in factor 1 items 9-17-20, in factor 2 items 7 and 24 and, finally, in factor 3 we omitted items 4-18-19-21. Notwithstanding, three items below the criteria were maintained, as they conceptually fit into the factor. Of the final 15 AAQ items, 5 belong to the Psychosocial Loss Factor, 6 to Physical Change Factor, and 4 to Psychosocial Growth Factor. This tree factor model explained 50.1% of the total variance. In conclusion, this study supports that AAQ has acceptable validity, confirming the composite reliability and the discriminant validity, but not the convergent validity. Through multi-group analysis, the invariance of the scale was confirmed. This validation is of pivotal importance once it allows measuring attitudes towards ageing in the Portuguese population, thus facilitating the prevention of ageism and the promotion of well-being across the lifespan.

## 1. Introduction

We are witnessing an exponential increase in the number of older people. This phenomenon, extensively described in gerontological literature [1], manifests itself worldwide. By 2050, according to WHO (2022), the world’s population of people aged 60 years and older will double (2.1 billion), and the number of persons aged 80 years or older is expected to triple between 2020 and 2050 to reach 426 million [2]. In Portugal, in 2021, the percentage of older adults (those aged 65 and over) was 23.7% of the total population. This age distribution led to an ageing ratio of 184.9 older adults per 100 young people [3]. Globally, it is estimated that the age group above 80 will be the fastest growing within the older adult population [4], so older people will occupy a considerable portion of the age pyramid. This growth requires us to reflect on and pay due attention to the increasingly evident specificities, needs, and demands of older individuals. Faced with the changes in their age group, they develop and reinvent themselves in an increasingly challenging modern daily life. Although the increase in the average life expectancy may be considered a social conquest and a challenge, it is, above all, a universal process that constitutes an unparalleled subjective experience for human beings. It is influenced by numerous factors, such as gender, physical condition, environment, social and behavioral determinants, psychological strategies, and culture [5,6]. However, it is not only essential to quantify the increasing proportion of older adults but to describe the quality of their experience. In this context, the construction, validation, and study of instruments that allow this type of assessment becomes especially relevant for research in Psychogerontology.

However, we can observe that: (a) most instruments to assess the subjective experiences of older subjects were not specifically built for this age group—quite on the contrary, the development of research in the area of psychological assessment of older people is recent [7]; (b) the discussion of the subjective experience of what it is to experience old age is made by subjects of other age groups, namely young adults and middle-aged adults, when it would be more relevant for the older adult subjects themselves to do so [8]; (c) although the experience of ageing is a cross-cultural phenomenon, it is largely influenced by each country’s culture, and not all instruments consider these aspects [9].

AAQ is about attitudes toward ageing and not only about experiences of being old. Attitudes toward ageing and old age are a prerogative of all people since they are essential for our identity development and definition. At this level, there is a notable shortage of psychological assessment instruments adapted to the Portuguese population.

In addition to the scientific community’s need to invest in the psychological assessment of older adults, there is an increase in the concerns related to the promotion of well-being in adulthood in the transition from a pathogenic paradigm, where the primary focus of interest is on disease etiology, to a salutogenic paradigm, which is dedicated to exploring the causes and consequences of health and positive functioning [10]. From the latter perspective, it is important to explore the factors that promote well-being rather than those that cause illness, since they act in a preventive and therapeutic way on mental and physical health [11,12,13].

In this realm, the United Nations General Assembly declared 2021–2030 the Decade of Healthy Ageing, and challenged WHO to lead the implementation of a global collaborative action to foster longer and healthier lives and reduce health inequities and improve the lives of older people, their families, and communities through collective action. One of these concerns is changing how we think, feel and act toward age and ageism [14]. To change attitudes toward ageing, we need to assess these attitudes.

Concerning research on well-being in old age, the literature also indicates that older people are no less satisfied with life than other age groups, regardless of the problems they may eventually have. Affective well-being evolves throughout life due to the increased ability to regulate emotions [15]. This ability depends, in part, on the attitudes that subjects have towards their ageing, which is greatly influenced by ageist stereotypes [16].

### 1.1. From Definition to the Assessment of Attitudes towards Ageing

From the perspective of the life cycle developmental approach, old age has been considered a vital stage with specific contours [17]. To better understand what this stage implies for the person experiencing it, it is important to understand the cognitive, affective, and behavioral processes it includes.

The construct, “attitude”, privileges access to the processes mentioned above because it translates the set of cognitive operations (beliefs/values), affections (basic emotions/feelings), and behaviors (actions) that are formed and transformed throughout the environment–subject interaction [18]. Although several studies address the developmental tasks and challenges the ageing population faces, few examine their attitudes toward ageing and their impact on the resolution of these tasks [19]. Changing how we think, feel, and act toward age and ageism is one of the main concerns of the Decade of Healthy Ageing 2021–2030 [14] declared by the United Nations General Assembly.

Attitudes toward ageing are an integral part of the culture of each society, reflecting how the older adult is observed and treated within it and conferring identity to the members of this age group, who, internalizing the attitudes they have learned from their socialization process, transmit them from generation to generation. Thus, from the point of view of socialization, the older adults themselves become agents of socialization that convey perceptions of the ageing process and of being old, just like their relatives, peers, institutions, and the media. Given the lack of instruments to assess the culturally imbued experience of becoming and being old, the AAQ instrument was built.

### 1.2. Attitudes to Ageing Questionnaire

The Attitudes to Ageing Questionnaire [9] is a self-report measure that aims to assess older people’s attitudes towards their ageing process and was developed based on current gerontological and psychometric knowledge.

The authors of the AAQ, Laidlaw, Power, and Schmidt, in collaboration with the WHOQOLD-OLD Group, argue that older people themselves are the best experts in this field and that they can and should be consulted regarding attitudes towards ageing. In fact, the initial step in developing the instrument was to set up a focus group, in Scotland, with thirty-five older adults with a mean age of 75 years (MIN = 62; MAX = 95), mostly (67%) female. This way, the general attitudes of these older people towards different aspects of their lives (past and present) were assessed. From the ideas generated by the group, five main conceptually relevant domains were built: the psychological domain; the physiological health domain; the social and interpersonal domain; the economic domain; and the social status and role of older adults. These domains could have a positive or a negative value. The experiment was then repeated in four groups with people from fifteen Scottish Day Care Centers. In addition to these groups, two groups of caregiver subjects were created: one composed of informal caregivers (family members) and the other of formal caregivers (health professionals).

In a second step, the authors used the “Delphi Technique”, which includes, on the one hand, the review of the existing literature on attitudes towards ageing and, on the other, the preliminary analysis of the topics and items generated. For the scale development, these contents were worked on by researchers from fifteen research centers.

The pilot version resulted in a forty-four-item scale with an equal number of positive and negative sentences. The items were grouped into the five domains generated by the focus groups: physical, psychological, social, economic, and role/social status. After translation and back-translation into the different languages by bilingual translators, the scale was applied to 1356 subjects from the 15 centers mentioned above. Each center collected a minimum of 60 participants with an equal number of subjects of each gender and was distributed into three age groups (60–69; 70–79; 80+). Subjects with a terminal illness, dementia, or other cognitive limitations that could significantly bias their perception of old age were excluded.

As a result of the analyses mentioned above, the final version of the AAQ was left with three subscales: the subscale of psychosocial losses, according to which old age is regarded primarily as a negative experience, where loss occurs at the psychological and social levels; the subscale of physical changes, which addresses aspects such as health, exercise, and the experience of the ageing process itself; the subscale of psychological growth, which focuses mainly on positive aspects such as wisdom and growth that reflect gains in the way subjects relate to themselves and others. Each of these subscales contains eight items and can be scored independently, giving a total score for each subscale. Note that in the subscale psychosocial losses, the items are inverted, so the higher the score, the more negative the perception of old age in this domain. In the remaining scales, the higher the score, the more positive the perception of old age will be.

It should be noted that an instrument such as the AAQ is an important contribution not only to the understanding of ageing but to the study of the ageism. Although the original instrument has been used essentially as a ‘self-report measure that aims to assess older people’s attitudes towards their ageing process and was developed based on current gerontological and psychometric knowledge’ [9], even Laidlaw, in the instructions of the AAQ, states: ‘This questionnaire asks you how you feel about growing older’ [9]. The author speaks about the process of growing old, and not about the development phase (old age).

Therefore, considering previous research, the current study looks to examine the psychometric properties of AAQ within Portuguese subjects. Though every new application of an instrument is, inherently, a contribution to its continual validation efforts, the development and validation of the AAQ adapted for the Portuguese language assumes an important contribution to a better understanding of attitudes to ageing. Hence, this study expects to help clear up how attitudes to ageing are perceived in the Portuguese context.

Additionally, the literature in the field of ageing stresses the need for more research, especially randomized clinical trials [20], which to this extent, justifies the effort to validate and adapt valid measuring instruments. In addition, some authors [21,22] have even suggested conducting studies on ageism in different cultural contexts. Therefore, the present study allows both to make the AAQ a stronger and more valid instrument and develop future studies on the ageing process and mental health variables [1,23]. As stated earlier, there is evidence that subjects with higher levels of self-perceived attitudes toward ageing have a greater creative adaptation. This seems to indicate that people’s attitudes towards ageing may be more positive when there are increased levels of personal psychological growth. These considerations may have various applications, ranging from the challenges inherent in life itself or even in crises, for example, the pandemic situation of COVID-19 [24,25].

Finally, in studies on ageing, the AAQ is the most used measuring instrument, so performing the validation of the Portuguese version is an important goal and a necessary work. Thus, this study aims to validate the AAQ in Portuguese. As proposed by the authors, we tested a three-factor scale, and by doing so, we intend to provide the scientific community and other professionals connected to the issue of ageing with a valid instrument to assess and diagnose their daily practice.

## 2. Materials and Methods

### 2.1. Participants

To obtain a heterogeneous sample, we included participants from different regions of Continental Portugal with a minimum age of 18 years, using convenience sampling. Thus, 400 literate subjects (142 male and 258 female), in the age spectrum between 18 and 93 years, participated in this study (M = 56.76, SD = 18.75). Concerning age, the value of the scale most often scored, reported by the mode, is 60 years old; the percentile 1 (25%) corresponds to 40 years old, the percentile 2 (50%) or median corresponds to 61 years old, and the percentile 3 (75%) corresponds to 71 years old. As for their place of residence, most come from rural and suburban areas (83.5%). A total of 45% of the subjects reported good subjective health and 31% fair, with an average of 3.66 (scores vary from 1 to 5). Men presented better subjective health than women.

### 2.2. Instrument

We used the Attitudes towards Ageing Questionnaire (AAQ) from Laidlaw, Power, and Schmidt [9], which comprises 24 items divided into three subscales of eight items each (Psychosocial Losses, Physical Change, and Psychological Growth) (Table 1). Respondents are asked to indicate their level of agreement on a 5-point Likert scale. The higher the score, the more positive the assessment of old age, except for the subscale Psychosocial Losses, where the items are inverted.

This instrument has been translated into many other languages. Examples are the Spanish version [26], the French version [23], and, more recently, the Malay Version [21], attesting to its interest and actuality. Recently, short versions of the questionnaire have been proposed [22].

### 2.3. Procedures

Participants were invited to the study voluntarily, and it was established that they would not receive any type of financial compensation. Before filling out the questionnaire, they were informed about the purpose and usefulness of the study and the importance of their participation. Finally, they were informed about how to answer and interpret the Likert scale. Afterward, each participant gave their approval and consent by checking a box on the questionnaire. A total of 400 questionnaires were collected, and after screening, they were deemed useful for data analysis. The average time to fill out the questionnaire was approximately 12 minutes.

In order to minimize discrepancies between the original and the Portuguese version the AAQ was submitted to a process of translation and back translation [27]. This process was conducted by a panel of experts, both in psychology and Portuguese fluent in English. After asses differences in meaning they concluded that the two scales are conceptually equivalent.

### 2.4. Statistical Analysis

As previously stated, the AAQ includes a negative wording factor. Thus, before performing the data analysis, we reversed the items in the “Psychosocial Loss” factor. Next, to assess the factorial validity of the AAQp, we performed a confirmatory factor analysis [28] using the maximum likelihood method [29]. 

To verify data distribution, we both calculated skewness and kurtosis coefficients and performed the Kolmogorov-Smirnoff test. To verify the inexistence of outliers, we calculated the Mahalanobis distance (D^2^) [30]. For the assessment of the model fit, we calculated the Chi-square statistic with Bollen Stein’s bootstrap method to correct non-normal and missing data (χ^2^); the Chi-square ratio by degrees of freedom (χ^2^/df), considering that χ^2^/df values lower than 5.0 or lower than 3.0 indicates an acceptable fit and good fit of the model to the data, respectively [30,31]. Other tests to evaluate the adequacy of the model were also performed, namely the GFI—Goodness of Fit Index, the CFI—Comparative Fit Index, and the TLI—Tucker-Lewis Index. This index must achieve 0.70; 0.80; and 0.90/0.95 to a good fit, very good fit, and excellent fit, respectively, of the model to the data. Moreover, the Root Mean Square Error of Approximation (RMSEA) was calculated. A good model fit is considered when this index is less than 0.06 and with a non-significant probability P(rmsea > 0.05) [29]. Z-test values were also used to verify the fit of the structural model and to test the relationships among the factors, considering factor loadings to be significant when Z ≥ 1.96 and *p* ≤ 0.05 [32]. We also assessed composite reliability and the internal consistency of the subscales. In the latter, we calculated Cronbach’s alpha [33], while we calculated the mean values of AVE—variance extracted to assess composite validity [34]. Specifically, we consider internal consistency and composite reliability to be good values when the values obtained are equal to or greater than 0.70, respectively. On the other hand, factors are considered reliable and valid when AVE values are equal to or greater than 0.50 [34]. Finally, we established that the discriminant validity is achieved when the AVE values for each factor exceed the squared correlation between that factor and the remaining squared values between that factor and the remaining factors [34]. Through multi-group analysis (using the Half-split step to divide the sample in two different and exclusive subsets), we also checked cross-validity. In this sense, the model invariance was checked by comparing the free model with the constrained models (fixed factor loadings and fixed variances/co-variances). The literature indicates for the Chi-square statistic that [35] factorial invariance is verified when there are no significant differences between the models (*p* > 0.05).

## 3. Results

**Figure 1:**
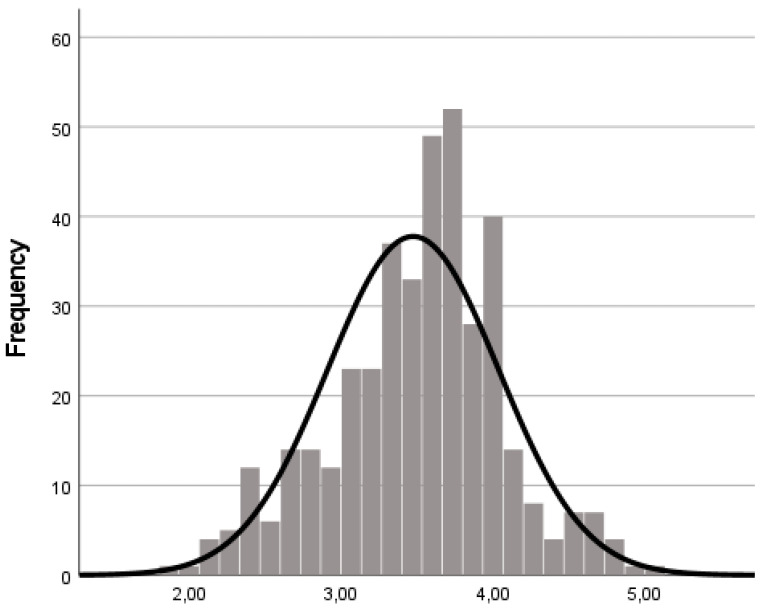
Histogram of AAQ scores (Mean = 3.47; DP = 0.56; Mode= 3.67; Median = 3.53; Percentile 25 = 3.13; Percentile 50 = 3.53; Percentile 75 = 3.80; *n* = 400).

Considering several tests, normal distribution could not be confirmed. Namely, skewness and kurtosis values are below 3 and 7 (Table 2), [31] and the Mardia coefficient indicated no multivariate distribution (Coefficient = 105.96) [30]. In this set (Figure 1) composed of 400 participants, the mean score of the responses is 3.47, the standard deviation is 0.56, reported by the mode the value of the scale most often scored is 3.67, the percentile 1 (25%) corresponds to 3.13, the percentile 2 (50%) or median corresponds to 3.53, and the percentile 3 (75%) corresponds to 3.80. Complementarily, the Kolmogorov-Smirnov test did not reveal the normality of distribution [K-S (439) = 0.085, *p* = 0.001)]. Thus, we used Bollen and Stine’s bootstrapping (B-S) [36] procedure to adjust the *p*-value of the Chi-square statistic.

The overall assessment of the structural model [χ^2^(249) = 838,185, B-S *p* < 0.001; χ^2^/df = 3.336; CFI = 0.73; GFI = 0.84; TLI = 0.70; RMSEA = 0.077; 90% CI [0.072–0.084] indicates a poor to acceptable fit of the model to the data. Not all values (namely, CFI and TLI) meet the recommended criterion (>0.80) for acceptable fit [36]. In addition, not all estimated factor loadings (Table 3) met the recommended cutoff point of 0.50 [35].

Thus, to improve the model fit, we decided to remove the items with loadings below 0.50, and if the items were theoretically relevant, the criterion below 0.45 loading was used. Namely, in the Psychosocial Loss factor, we removed item 9 (“I find it more difficult to talk about my feelings as I get older”) and item 17 (“As I get older I find it more difficult to make new friends”) since they had a factor loading of 0.28 and 0.31, respectively. In the Physical Changes factor, items 7 (“It is important to take exercise at any age“) and 24 (“I keep myself as fit and active as possible by exercising“) were removed, since they had a factor loading of 0.30 and 0.45, respectively. In the Psychological Growth factor, we removed items 4 (“Wisdom comes with age”), 18 (“It is important to pass on the benefits of my experience to younger people”), 19 (“I believe my life has made a difference”) and 21 (“I want to give a good example to younger people”), since they had a factor loading of 0.25, 0.40, 0.38 and 0.42, respectively. Further, the RMSEA demonstrated an acceptable fit [10,11].

In the version after the removal of the items (see Table 3), the overall assessment of the structural model [χ^2^(87) = 287.318, B-S *p* < 0.001; χ^2^/df = 3.303; CFI = 0.87; GFI = 0.90; TLI = 0.84; RMSEA = 0.076; 90% CI [LO = 0.067—HI = 0.087] indicates an acceptable model fit to the data. The values of CFI and TLI meet the recommended criterion (>0.80) for acceptable fit, and the GFI value meets the recommended criterion for good fit. RMSEA also demonstrated a good adjustment [32,33].

It should be noted that in this model, factor 1 (“Psychosocial loss”), item 20 (“I don’t feel involved in society now that I am old”) did not meet adequate factor loading (≧0.50) nor adequate individual reliability (R^2^ ≧ 0.25), scoring 0.16, and so we decided to remove it from the model, notwithstanding; the remaining items displayed adequate individual reliability ranging between 0.45 and 0.83. The Z-test values also indicated statistical significance ranging between 7.27 and 12.38 [33]. Regarding the composite reliability of the constructs, we can say that in both the Psychosocial Loss and the Physical Change factors, the recommended cut-off values were reached, with values between 0.77 and 0.72, respectively. It should be added that the same did not happen for the psychological growth construct, which did not reach the minimum recommended value for this test since it only reached a value of 0.65. Thus, it is not possible to state that the scale fully meets the criteria for composite reliability [37]. Furthermore, it was also not possible to fully verify convergent validity, since the AVE values are below 0.50, the minimum recommended value for this index [34,38]. Despite some limitations regarding composite reliability and convergent validity, the scale showed robustness and very good reliability from the global point of view, since Cronbach’s alpha reached an internal consistency value of 0.84. Moreover, all subscales also demonstrated good reliability, namely, the Psychosocial Loss subscale reached a Cronbach’s alpha value of 0.77, the Physical Change subscale reached a Cronbach’s alpha value of 0.72, and the Psychosocial Growth subscale reached a Cronbach’s alpha value of 0.70.

**Figure 2:**
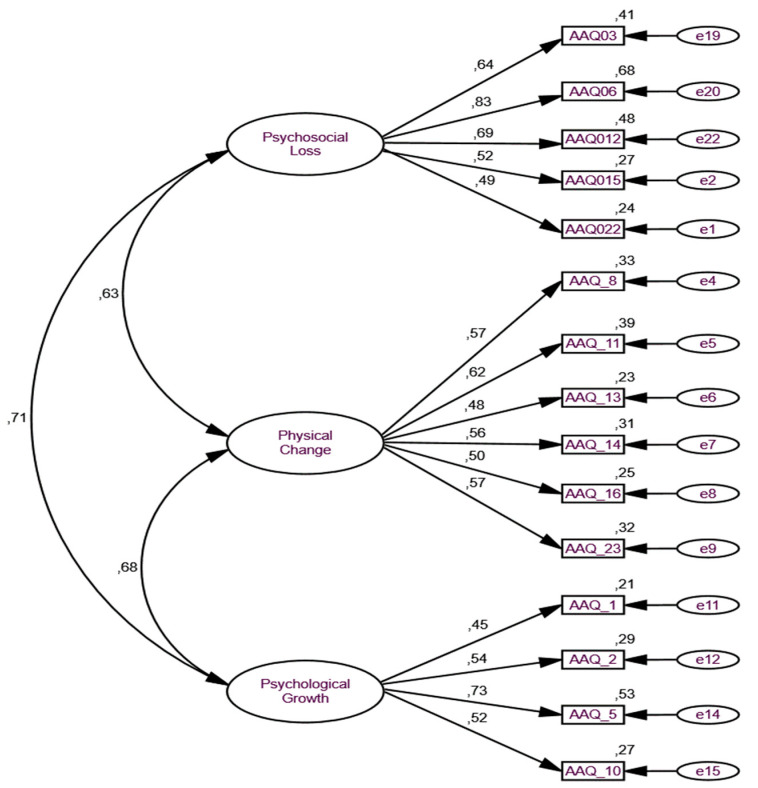
CFA of the re-specified 3-factorial AAQp.

The parallel analysis obtained through the communalities also revealed satisfactory values, and with only two exceptions, all items reached the cutoff value of 0.40. Additionally, the total scale revealed no statistically significant differences between genders (*p* = 0.139). Namely, the scale revealed an overall mean of 3.47 (SD = 0.56), and between genders, the values were 3.44 (SD = 0.61) for women and 3.53 (SD = 0.46) for men. Figure 2 shows the re-specified AAQ model with 15 items.

Table 4 presents both the AVE (average variance extracted) values and factors R^2^ (squared correlations). Given that none of the R^2^ of the factors exceeded the AVE values, the discriminant validity was assumed.

### Cross-Validity

Using multi-group analysis to check for cross-validity, we studied the invariance of the model (Table 5). As stated, we used a half-split procedure to perform the multi-group analysis studying the differences between the two different but equivalent samples. Thus, it is in Sample 1, which corresponds to 191 subjects that took part in the study and in Sample 2, which corresponds to 209 subjects that took part in the study). As shown in Table 5, the fit of the free model [Model 1: χ^2^ (174) = 377.220; PCFI = 0.72; PGFI = 0.64; CFI = 0.86; GFI = 0.88; RMSEA = 0.05] demonstrated an acceptable fit [32]. Inspecting the model where the variance is fixed [Model 2: χ^2^ (186) = 381.914 (B-S *p* < 0.967); PCFI = 0.77; PGFI = 0.68; GFI = 0.88; CFI = 0.87; RMSEA = 0. 05] and the model where residuals are fixed [Model 3: χ^2^ (192) = 388.158 (B-S *p* < 0.396); PCFI = 0.79; PGFI = 0.70; GFI = 0.88; CFI = 0.87; RMSEA = 0.05], it is found that both have acceptable model fit to the data. The Chi-square (χ^2^) statistics revealed that there are statistically significant differences between Model 1 and Model 2 (χ^2^dif (12) = 4.7; B-S *p* = 0.967) or Model 1 and Model 3 (χ^2^dif (18) = 10.9; B-S *p* = 0.730). We thus consider that the model invariance is demonstrated when compared in two different samples, i.e., the results demonstrated that the factorial structure of the AAQp does not differ between two different samples [32,35].

## 4. Discussion

The purpose of this work was to study the characteristics of the Portuguese version of the Attitudes to Ageing Questionnaire in a 3-first-order factors version (Psychosocial Losses, Physical Change and Psychological Growth), following the suggestions of the original authors [9,22].

Old age continues to be portrayed mainly as a negative experience involving psychological and social loss [2]. One in every two people are ageists against older people, and in Europe, younger people report more perceived ageism than other age groups. Many misleading myths and misconceptions about ageing are treated as facts and truths, although they are false. Nevertheless, they impact personal well-being and are responsible for the low adherence of older adults to many social and health interventions.

Ageism is a compound of stereotypes (how we think), prejudices (how we feel), and discrimination (how we act) towards others or ourselves based on age, informing what we call attitudes towards age, the ageing process and the older adults.

Therefore, measuring attitudes towards ageing, especially knowing the opinions of those in the last phase of the life cycle, is a more realistic approach than characterizing a phase of the life cycle according to ageist projections and stereotypes. Bringing gender, cohort, historical and cultural perspectives to the equation allows us to have a much more variegated approach to the ageing process and experience.

Our option to use a sample of adults aged 18 and over is related to increasing the predictive value of the scale. In this sense, our sample comprised adults between 18 and 93 years old. The possibility of getting to know and, consequently, being able to intervene with younger adults is, as we have observed, fundamental in this area.

In our study, AAQp emerges as a short and valid instrument when one intends to evaluate attitudes toward ageing. As found in previous studies, both with the English [9] and non-English versions [13,21], AAQp demonstrated overall acceptable to good reliability (with Cronbach’s alphas ranging from 0.77 to 0.65). Furthermore, composite reliabilities were achieved but not convergent validity, as the AVE values were lower than 0.50.

We used the original tridimensional model. However, some items were removed as in other versions [13]. Namely, in the Psychosocial Loss factor, items 9 (“I find it more difficult to talk about my feelings as I get older”) and 17 (“As I get older, I find it more difficult to make new friends”) were removed. In the Physical Changes factor, items 7 (“It is important to take exercise at any age“) and 24 (“I keep myself as fit and active as possible by exercising“) were removed. In the Psychological Growth factor, items 4 (“Wisdom comes with age”), 18 (“It is important to pass on the benefits of my experience to younger people”), 19 (“I believe my life has made a difference”) and 21 (“I want to give a good example to younger people”) were removed. Because the model displays better adjustment indexes without these items, we suggest their withdrawal from the scale. As said, this suggestion is due to two reasons. First, these items show poor factor loading (below-recommended cut-off point) [24]. Secondly, the modification indices (MI) suggested some error correlations, but we could not confirm that these errors, when correlated, significantly improve the model fit. More to the point, examining the content of these items made it clear that they have some degree of redundancy, so they were removed from the model.

As we have observed, other versions of the AAQ did not find the same low loadings or conceptual overlaps that we indicated. This may be because these items were translated to Portuguese, and in some cases, they may have an ambiguous interpretation or somehow inadequate interpretation by the youngsters. In future studies, the revision of these items should be considered.

Despite these aspects, we believe that there is sufficient evidence to support the factor structure that we propose in our study. The 3-factor structure of the AAQ with 15 items proved to meet both construct validity and content validity, and the results of the internal consistency demonstrated that the reliability of the scale is acceptable. Moreover, the confirmatory factor analysis revealed that the scale has an acceptable adjustment to the data. Evidence was found to support the confirmation of composite reliability and discriminant validity [29]. Although convergent validity has not been fully confirmed, we think the final structure offers an acceptable structure to consider the AAQ a reliable measure to assess attitudes toward ageing.

The proposed validation provides researchers and professionals with an instrument that allows better management of their daily practice. Thus, the adapted version of the AAQ may represent a very useful tool for the important task of assessing programs aimed at the development of attitudes towards ageing, as well as for the development of research with a multidimensional design, i.e., including other variables related to the understanding of the phenomenon of ageing in a cultural and Portuguese-speaking context. Looking forward, we can state that it is a step that will provide a theoretical framework facilitating the planning and promotion of psycho-educational models toward ageing. This last aspect is particularly important in promoting quality of life and mental health.

## 5. Conclusions

This study provides supporting evidence for the Portuguese version of the AAQ. In particular, the validity and reliability of the tri-factor version of the AAQ proposed by Laidlaw et al. [9] was confirmed. Namely, the original scale proposes three factors (Psychosocial Losses, Physical Change and Psychological Growth). 

As a result of the confirmatory factor analysis, both the Psychosocial Losses (items 3, 6, 12, 15, 22) and the Physical Changes factors (8, 11, 13, 14, 16, 23) were reduced to 6 items each, while the Psychological Growth (items 1, 2, 14, 15) factor was reduced to 4 items. Thus, the final scale has 15 items. Readers interested in the Portuguese version of the AAQ can consult the Appendix A at the end of this article.

Moreover, using the measure of attitudes towards ageing benefits development theory and may help innovate anti-ageism-based interventions [22,39]. This is of urgent importance in the face of demographic changes. All countries face major challenges in ensuring their health and social systems are ready to embrace the demographic shift. Thus, correctly assessing the variables that impact this process is fundamental. Complementary, through the measure presented here, there is the possibility of carrying out several studies, such as studying the relationship between attitudes towards ageing and creative adaptability in Lusophone communities and comparing it with other linguistic and cultural spaces [21,23], given that each new application in a different context represents a contribution to improving the theoretical value of the research domain [14]. In this sense, understanding attitudes toward ageing can help prevent its negative impact throughout our life. Studies on ageing and ageism are usually applied and evaluated within communities over 60 years of age. This study helps also to expand and open new paths in the assessment of ageism, as the validity of the AAQ was confirmed in a different age context than the usual one.

In conclusion, the questionnaire on attitudes towards ageing reveals properties and psychometric qualities acceptable among young, middle-aged and older adults.

## 6. Limitation and Future Research

As with other research, this study includes some limitations and strategic options. We chose to use a sample of adults between 18 and 93 because we consider that the conceptions about age affecting the experience of old age start to be built many decades before reaching 60. Half of our participants are below 60 years of age. Therefore, future studies should increase the number of older participants. Although we have made this choice, we understand and support Laidlaw’s and colleagues [9,22] claim that “older people are experts to be consulted”. Actually, this was one of the main reasons for creating the ageing attitude scale. Although important insights into the ageing experience can only be derived from working with older adults, the importance of measuring attitudes towards ageing in younger individuals brings valuable information, making it easier to prevent negative attitudes towards growing older themselves or their attitudes towards older people and therefore minimizing malaise and anguish in the subjective experience of the older individuals. We cannot forget that entering old age is a cultural construction that varies with the culture and historical period in which we are embedded.

Due to the amplitude of ages of this study, we did not include, such as in other validation studies [26], participants aged 60 years and older from institutions such as community, residential, and primary care centers and associations for the mentally ill and dementia. Considering that the participants are from non-clinical settings, caution concerning using this tool is recommended when assessing levels of attitudes towards ageing in these settings.

## Figures and Tables

**Table 1:** Dimensions, description, and corresponding items from the original tri-factorial version of the AAQ [9].

Factors	Description	Items
Psychosocial Loss	*Measures the perceived negative experiences of ageing and functions as a proxy for negative attitudes to ageing, where old age is observed primarily as a negative experience involving psychological and social loss.*	*3-6-9-12-15-17-20-22*
Physical Change	*Focuses on items primarily related to health and the* *experience of ageing itself; therefore, a subjective individualized psychological perspective on health is assessed.*	*7-8-11-13-14-16-23-24*
Psychological Growth	*Explicitly positive and could be summarized as ‘Personal* *Wisdom’, as it recognizes a lifespan development perspective on ageing as viewed by the individual.*	*1-2-4-5-10-18-19-21*

**Table 2:** Descriptive statistics of the 3-factors, AAQp.

Item	M	SD	Min	Max	Ass.	Kurt.
***Psychosocial Loss***						
3. Old age is a time of loneliness	3.26	1.07	1	5	−0.278	−0.755
6. Old age is a depressing time of life	3.46	1.12	1	5	−0.595	−0.458
9. I find it more difficult to talk about my feelings as I get older	3.53	1.05	1	5	−0.431	−0.729
12. I see old age mainly as a time of loss	3.41	1.01	1	5	−0.251	−0.789
15. I am losing my physical independence as I get older	3.35	1.05	1	5	−0.194	−0.872
17. As I get older, I find it more difficult to make new friends	3.26	1.16	1	5	−0.134	−1.11
20. I don’t feel involved in society now that I am older	3.73	0.99	1	5	−0.674	−0.162
22. I feel excluded from things because of my age	3.86	0.92	1	5	−0.687	−0.006
***Physical Change***						
7. It is important to exercise at any age	4.55	0.59	2	5	−1.33	2.46
8. Growing older has been easier than I thought	3.20	0.94	1	5	−0.252	−0.539
11. I do not feel old	3.60	1.10	1	5	−0.720	−0.243
13. My identity is not defined by my age	3.82	1.07	1	5	−1.10	0.632
14. I have more energy now than I expected for my age	3.23	0.97	1	5	−0.142	−0.672
16. Problems with my physical health do not hold me back from doing what I want to do	3.21	1.07	1	5	−0.296	−0.811
23. My health is better than expected for my age	3.20	0.96	1	5	−0.296	−0.143
24. I keep myself as fit and active as possible by exercising	3.68	1.01	1	5	−0.773	0.135
***Psychological Growth***						
1. As people get older, they are better able to cope with life	3.67	0.90	1	5	−0.844	0.508
2. It is a privilege to grow old	3.62	1.5	1	5	−0.568	−0.439
4. Wisdom comes with age	3.60	0.97	1	5	−0.620	−0.216
5. There are many pleasant things about growing older	3.54	0.96	1	5	−0.813	0.082
10. I am more accepting of myself as I have grown older	3.69	0.87	1	5	−0.612	0.034
18. It is important to pass on the benefits of my experience to younger people	3.87	0.81	1	5	−0.595	0.370
19. I believe my life has made a difference	3.66	0.82	1	5	−0.298	0.044
21. I want to give a good example to younger people	4.09	0.70	1	5	−0.695	1.44

Note: M = mean; SD = Standard deviation; Min = minimum; Max = maximum; Ass. = Asymmetry; Kurt. = Kurtosis.

**Table 3:** Factor loadings, Z-values, communalities of the original scale with 24 items, and Cronbach alpha and composite reliability of the re-specified model of the AAQp with 15 items.

Factors/Items	λ	Z-Value	C	α	*CR*
***Psychosocial Loss***				0.77	00.78
3. Old age is a time of loneliness	0.64	9.10	0.48		
6. Old age is a depressing time of life	0.77	11.81	0.62		
9. (-) I find it more difficult to talk about my feelings as I get older	0.28	4.99	0.23		
12. I see old age mainly as a time of loss	0.69	11.05	0.45		
15. I am losing my physical independence as I get older	0.55	9.24	0.42		
17. (-) As I get older, I find it more difficult to make new friends	0.31	5,49	0.37		
20. (-) I don’t feel involved in society now that I am older	0.45	7.70	0.39		
22. I feel excluded from things because of my age	0.55	9.15	0.40		
***Physical Change***				0.72	0.72
7. (-) It is important to exercise at any age	0.30	5.05	0.13		
8. Growing older has been easier than I thought	0.54	5.26	0.47		
11. I don’t feel old	0.61	5.37	0.40		
13. My identity is not defined by my age	0.48	5.08	0.31		
14. I have more energy now than I expected for my age	0.54	4.86	0.44		
16. Problems with my physical health do not hold me back from doing what I want to do	0.51	5.01	0.29		
23. My health is better than expected for my age	0.58	5.00	0.43		
24. (-) I keep myself as fit and active as possible by exercising	0.45	4.84	0.27		
***Psychological Growth***				0.70	0.65
1. As people get older, they are better able to cope with life	0.49	5.11	0.28		
2. It is a privilege to grow old	0.52	7.41	0.26		
4. (-) Wisdom comes with age	0.25	3.52	0.35		
5. There are many pleasant things about growing older	0.66	7.94	0.41		
10. I am more accepting of myself as I have grown older	0.52	7.53	0.29		
18. (-) It is important to pass on the benefits of my experience to younger people	0.40	4.72	0.56		
19. (-) I believe my life has made a difference	0.39	5.11	0.49		
21. (-) I want to give a good example to younger people	0.42	5.14	0.55		

Notes: λ = factor loading; Z-value = Critical ratio; C = Communalities; α = Cronbach alpha; CF = Composite reliability; (-) items removed from the re-specified model.

**Table 4:** Mean (M), standard deviation (SD), and squared factor correlations.

Factor	AVE	Correlations Matrix		
		1	2	3
1. Psychosocial loss	0.42	1.00		
2. Physical change	0.30	0.26 **	1.00	
3. Psychological growth	0.32	0.17 **	0.20 **	1.00

Note. ** *p* < 0.01.

**Table 5:** Results of the CFA multi-group analysis of the AAQp 3-factor.

Models	χ^2^	gl	∆χ^2^	∆gl	B-Sp	PCFI	PGFI	GFI	CFI	RMSEA	IC 90%
Model 1	377.220	174	---	---	---	0.72	0.64	0.88	0.86	0.05	[0.047, 0.062]
Model 2	381.914	186	4.7	12	0.967	0.77	0.68	0.88	0.87	0.05	[0.051, 0.065]
Model 3	388.158	192	10.9	18	0.730	0.79	0.70	0.88	0.87	0.05	[0.043, 0.058]

Note. Sample 1: *n* = 191; Sample 2: *n* = 209.

**Figure A1:**
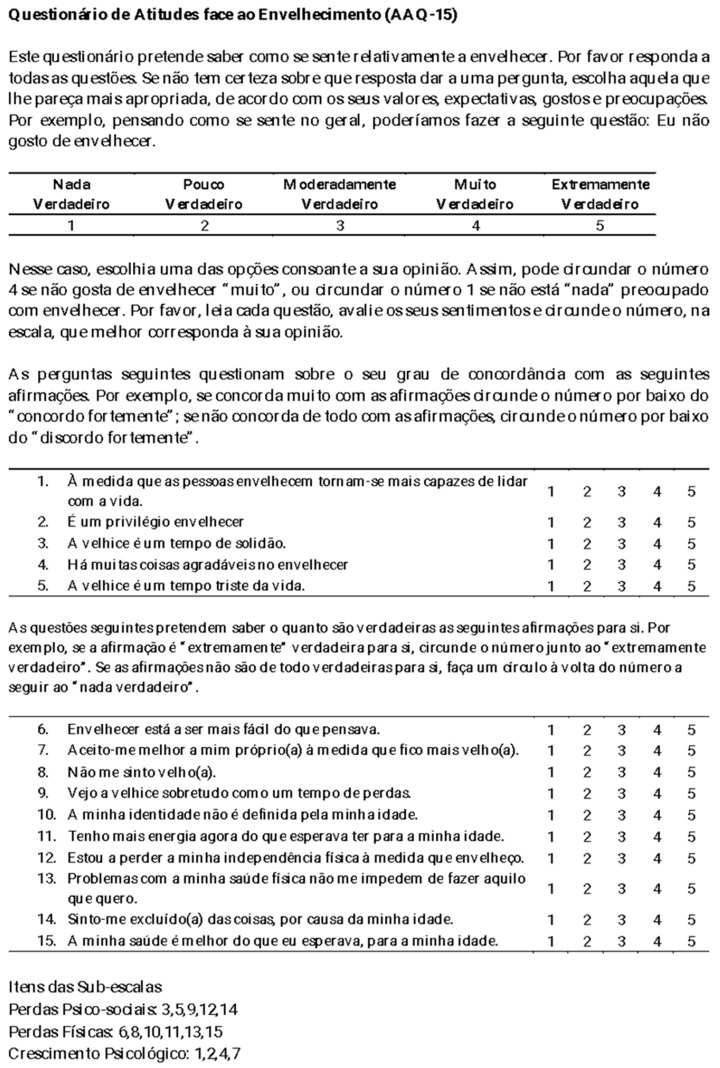
Portuguese Version of the Ageing Attitudes Questionnaire.

## Data Availability

Yes. Contact the authors.
